# Disease burden and treatment satisfaction in patients with prurigo nodularis in Japan

**DOI:** 10.1111/1346-8138.17045

**Published:** 2023-12-08

**Authors:** Hiroyuki Murota, Kazuhiko Arima, Takuo Yoshida, Hiroyuki Fujita

**Affiliations:** ^1^ Department of Dermatology Nagasaki University School of Medical Sciences Nagasaki Japan; ^2^ Sanofi K.K. Tokyo Japan

**Keywords:** burden of disease, cross‐sectional study, prurigo nodularis, surveys and questionnaires, treatment satisfaction

## Abstract

Prurigo nodularis (PN) is a chronic inflammatory skin disorder with a high disease burden. In this cross‐sectional, web‐based survey, Global Questions (GQ), the Numerical Rating Scales (NRS) for pruritus, burning sensation and sleep disturbance, the Short‐Form‐8 (SF‐8) Health Survey, Dermatology Life Quality Index (DLQI), Patient Health Questionnaire 9 (PHQ‐9), Work Productivity and Activity Impairment (WPAI), and Treatment Satisfaction Questionnaire for Medication–9 (TSQM‐9) scores were used to assess the current disease burden and treatment satisfaction among patients with PN in Japan. In total, 97 patients were included (55.7% male, median age 51 years, median duration of PN 36 months). Based on GQ scores, 35.1% of patients had mild disease, 50.5% moderate, and 14.4% severe disease. Disease burden increased as the severity of PN increased, as indicated by worsening of pruritus NRS scores and quality of life (DLQI, PHQ‐9, WPAI presenteeism, work productivity loss, and activity impairment scores). Patients with comorbid atopic dermatitis (AD) also had more intense pruritus than those without AD. Mean ± standard deviation TSQM‐9 scores for effectiveness, convenience, and global satisfaction were 54.7 ± 18.1%, 62.4 ± 15.2%, and 57.4 ± 15.9%, respectively. TSQM‐9 scores were lowest in patients receiving the most intensive guideline‐directed treatment (i.e., topical corticosteroids + systemic oral corticosteroids or cyclosporine), highlighting an unmet need for more effective treatment options for patients with PN. In summary, Japanese patients with PN reported increased disease burden and reduced treatment satisfaction with increased disease severity, despite the use of guideline‐recommended therapies.

## 1 INTRODUCTION

Prurigo nodularis (PN) is a chronic, inflammatory skin disease characterized by intensely pruritic, hyperkeratotic, and symmetrically distributed nodules.^1^, ^2^, ^3^ The estimated prevalence of PN was reported as 72/100 000 adults aged 18–64 years in an epidemiological study in the United States.^1^ In a nationwide Japanese Dermatological Association study of 67 448 dermatology patients, 1229 (1.8%) had prurigo,^4^ a condition that includes PN, prurigo chronic multiformis, or prurigo (not otherwise specified).^2^ Although its pathogenesis is not fully understood, significant dysregulation of immune cells and neuronal pathways may play an important role in PN development.^5^

The burden of disease among patients with PN is reportedly higher than that of other forms of inflammatory dermatosis, with a higher incidence and intensity of pruritus and a greater impact on health‐related quality of life (HRQoL).^6^ According to a systematic review of 13 studies, PN is associated with reduced HRQoL, largely because of persistent pruritus as well as skin lesions and sleep disturbance.^7^ In addition, PN may increase the disease burden of associated medical conditions, including mental health disorders, cardiovascular disease, chronic kidney disease, chronic obstructive pulmonary disease, chronic hepatitis C, HIV infection, and atopic dermatitis (AD).^1^, ^8^ A large, web‐based survey of patients with PN in Europe reported that pruritus symptoms were associated with the greatest disease burden; other frequently reported sensory symptoms included burning, stinging, and pain.^9^ In this European survey, 56.8% of patients reported a lack of treatment satisfaction in the previous 6 months.^10^ However, the range and degree of the disease burden and how this burden impacts treatment satisfaction in Japanese patients with PN has not been investigated.

In this study, we conducted an online questionnaire of adult patients with PN in Japan who were diagnosed and treated by dermatologists, to assess their current disease burden and treatment satisfaction.

## 2 METHODS

### 2.1 Study design

A web‐based, cross‐sectional, observational study of patients with PN and their treating dermatologists was conducted in Japan between April 1 and May 31, 2022 (UMIN‐Clinical Trials Registry: UMIN000047643).

Dermatologists were recruited by Plamed Inc. (a market research company with approximately 53 000 registered physicians); patients with physician‐diagnosed PN who were referred by these dermatologists were eligible for study inclusion. All responses received were anonymized and analyzed by Social Survey Research Information Co., Ltd (Tokyo, Japan). This article reports the results of the survey in patients with PN; the results from the dermatologist survey will be published separately elsewhere.

The study was conducted in accordance with the principles of the Declaration of Helsinki and the Ethical Guidelines for Life Sciences and Medical Research Involving Human Subjects, issued by the Ministry of Health, Labour and Welfare, the Ministry of Education, Culture, Sports, Science and Technology, and the Ministry of Economy, Trade and Industry in Japan. The study protocol was approved by the Ethics Review Board of the Medical Corporation Tohkei‐kai Kitamachi Clinic, Tokyo, Japan, on March 16, 2022 (study no. OBS17567), in accordance with local regulations, including data protection laws.

### 2.2 Patients

Respondents aged >20 years with physician‐diagnosed PN and who lived in Japan were invited to participate. They had to have a disease duration of ≥3 months and have visited a medical institution for PN in the past 6 months. All respondents provided their informed consent to participate in the study before starting the questionnaire. Those who provided inappropriate responses were excluded from the analysis, and the resulting respondents are hereafter referred to as patients.

### 2.3 Study objectives

The primary objective was to describe the disease burden among patients with PN using multiple patient‐reported outcomes (PROs) for global severity; extent of weekly pruritus, burning sensation, and sleep disturbance; general and skin‐specific quality of life (QoL); depressive status; work productivity and activity impairment; and treatment satisfaction. We also described each PRO score by disease severity, presence of AD, and treatment category. Treatment categories were defined following the 2020 Japanese guidelines for prurigo according to increasing treatment intensity as follows: line 1 (L1), over‐the‐counter (OTC) skin care only; L2, topical corticosteroid (TCS) therapy ± antihistamines ± ultraviolet B (UVB) phototherapy; L3, TCS therapy + adjunctive therapy (including combined used of local corticosteroid injection, topical therapy with heparinoid ointment [occlusive application] vitamin D3 analogs, antipruritic ointment, capsaicin ointment, liquid nitrogen, neurotropin [extracted fluid from the inflamed skin of rabbits inoculated with vaccinia virus] reserpine, gabapentin, pregabalin, or Chinese herbal medicine); or L4, TCS therapy + systemic oral corticosteroid (OCS) therapy, or cyclosporine.^2^

The secondary objective was to describe treatment satisfaction using the Treatment Satisfaction Questionnaire for Medication – 9 items (TSQM‐9) score. We also evaluated TSQM‐9 scores according to patient subgroups (disease severity, presence of AD, and treatment category) and the correlation between TSQM‐9 and the above‐listed PROs.

### 2.4 Patient‐reported outcomes

The questionnaire included items regarding the patients' demographic and disease characteristics, communication with physicians, and the following seven PROs: (1) Global Question (GQ; 0–4 points),^11^ which is a 5‐point scale that assessed the severity of PN in the past 7 days (scores of 0–1 = mild; 2 = moderate; and 3–4 = severe); (2) the Numerical Rating Scales (NRS) for pruritus, burning sensation, and sleep disturbance (0–10 points), which indicated the intensity (severity) of symptoms in the past 7 days (scores of 0 = none; 1–3 = mild; 4–6 = moderate; 7–9 = severe; 10 = very severe); (3) the 8‐item Short Form Health Survey (SF‐8; 0–100 points),^12^, ^13^ which measured HRQoL over the past month in two standardized domains (physical health summary and mental health summary; higher scores indicate better HRQoL); (4) the Dermatology Life Quality Index (DLQI; 0–30 points),^14^ which assessed the impact of the disease on HRQoL (scores of 0–1 = no effect; 2–5 = small effect; 6–10 = moderate effect; 11–20 = large effect; 21–30 = extremely large effect); (5) the Patient Health Questionnaire 9 (PHQ‐9; 0–27 points),^15^, ^16^ which assessed depression severity over the past month (scores of 0–4 = none; 5–9 = minor to mild; 10–14 = moderate; 15–19 = moderate to severe; 20–27 = severe); (6) Work Productivity and Activity Impairment (WPAI; 0%–100%),^17^ which evaluated work and productivity impairment over the past 7 days across four domains: absenteeism (lost working hours due to PN), presenteeism (work impairment due to PN), work productivity loss, and activity impairment; and (7) TSQM‐9 (0%–100%),^18^ which included nine questions that evaluated effectiveness, convenience, and global satisfaction domains with higher scores indicating higher satisfaction in each domain.

### 2.5 Statistical analysis

The study outcomes were assessed using summary statistics (mean, standard deviation [SD], median, interquartile range, and minimum/maximum values). Missing data were not imputed.

Statistical analysis for age and the duration of PN were conducted using the Kruskal‐Wallis test, and for sex using the Fisher's exact test. For disease burden outcomes, the Steel–Dwass test was used for post‐hoc pairwise comparisons between three or more subgroups after significant Kruskal‐Wallis test, and the Mann–Whitney *U* test was used for pairwise comparisons between two subgroups (*p* < 0.05 indicated statistical significance).

The correlation between TSQM‐9 and GQ, NRS, SF‐8, DLQI, PHQ‐9, and WPAI scores was determined by calculating the Spearman's rank correlation coefficient (*r*_s_; where an *r*_s_ of ±0.4 to ±1.0 was considered to indicate a meaningful positive or negative correlation) and the *p*‐value for the pairwise comparison.

The data processing and statistical analysis were performed using Microsoft Excel 2013/2016; BellCurve Hideyoshi Dplus, version 1.12 (Social Information Service Co., Ltd.); Excel Statistics, version 3.23 (Social Information Service Co., Ltd.); and R software, version 4.1.2,^19^ for correlation.

## 3 RESULTS

### 3.1 Study population

Of 117 patients with physician‐diagnosed PN, valid responses were obtained from 97 patients; 20 patients did not provide consent and were excluded from the analysis.

The 97 patients in the study cohort 55.7% were male with a median age of 51 years, and a median PN duration of 36 months (Table 1). Based on their GQ score, 64.9% of patients had moderate‐to‐severe disease severity in the past 7 days. Compared with patients with mild or moderate disease, those with severe disease were slightly older and a greater proportion were male (Table 1). Patients with comorbid AD (*n* = 28; 28.9%) had a longer mean duration of PN than those without AD (107.6 vs. 50.5 months; Table S1).

**TABLE 1:** Patient demographics and baseline characteristics overall and by disease severity.

Item	Study cohort *N* = 97	Disease severity (GQ score)			
		Mild (0–1) *n* = 34	Moderate (2) *n* = 49	Severe (3, 4) *n* = 14	*p*‐value
Sex, *n* (%)					0.224^a^
Male	54 (55.7)	15 (44.1)	30 (61.2)	9 (64.3)	
Female	40 (41.2)	18 (52.9)	17 (34.7)	5 (35.7)	
Unknown/no answer	3 (3.1)	1 (2.9)	2 (4.1)	0	
Age, median (min, max), years	51 (23, 78)	52 (23, 76)	50 (26, 73)	58 (40, 78)	0.187^b^
PN duration, median (min, max) months	36 (4, 420)	36 (6, 420)	36 (6, 420)	32 (4, 120)	0.483^b^
Mean ± SD GQ score	2.8 ± 0.8	1.0 ± 0.2	2.0 ± 0.0	3.3 ± 0.5	
Medical history, *n* (%)					
Atopic dermatitis	28 (28.9)	8 (23.5)	16 (32.7)	4 (28.6)	
Allergic rhinitis	19 (19.6)	7 (20.6)	9 (18.4)	3 (21.4)	
Urticaria	13 (13.4)	4 (11.8)	6 (12.2)	3 (21.4)	
Diabetes	13 (13.4)	2 (5.9)	10 (20.4)	1 (7.1)	
Asthma	7 (7.2)	4 (11.8)	3 (6.1)	0	
Liver dysfunction	4 (4.1)	0	1 (2.0)	3 (21.4)	
Metal hypersensitivity	2 (2.1)	1 (2.9)	1(2.0)	0	
Blood disorder	2 (2.1)	1 (2.9)	1 (2.0)	0	
Malignancy	0	–	–	–	
Renal dysfunction	0	–	–	–	
HIV infection	0	–	–	–	
Pulmonary diseases	0	–	–	–	
Unspecified	41 (42.3)	16 (47.1)	19 (38.8)	6 (42.9)	

Abbreviations: GQ, Global Question; max, maximum; min, minimum; PN, prurigo nodularis; SD, standard deviation.

^a^ Fisher's exact test.

^b^ Kruskal–Wallis test.

**FIGURE 1:**
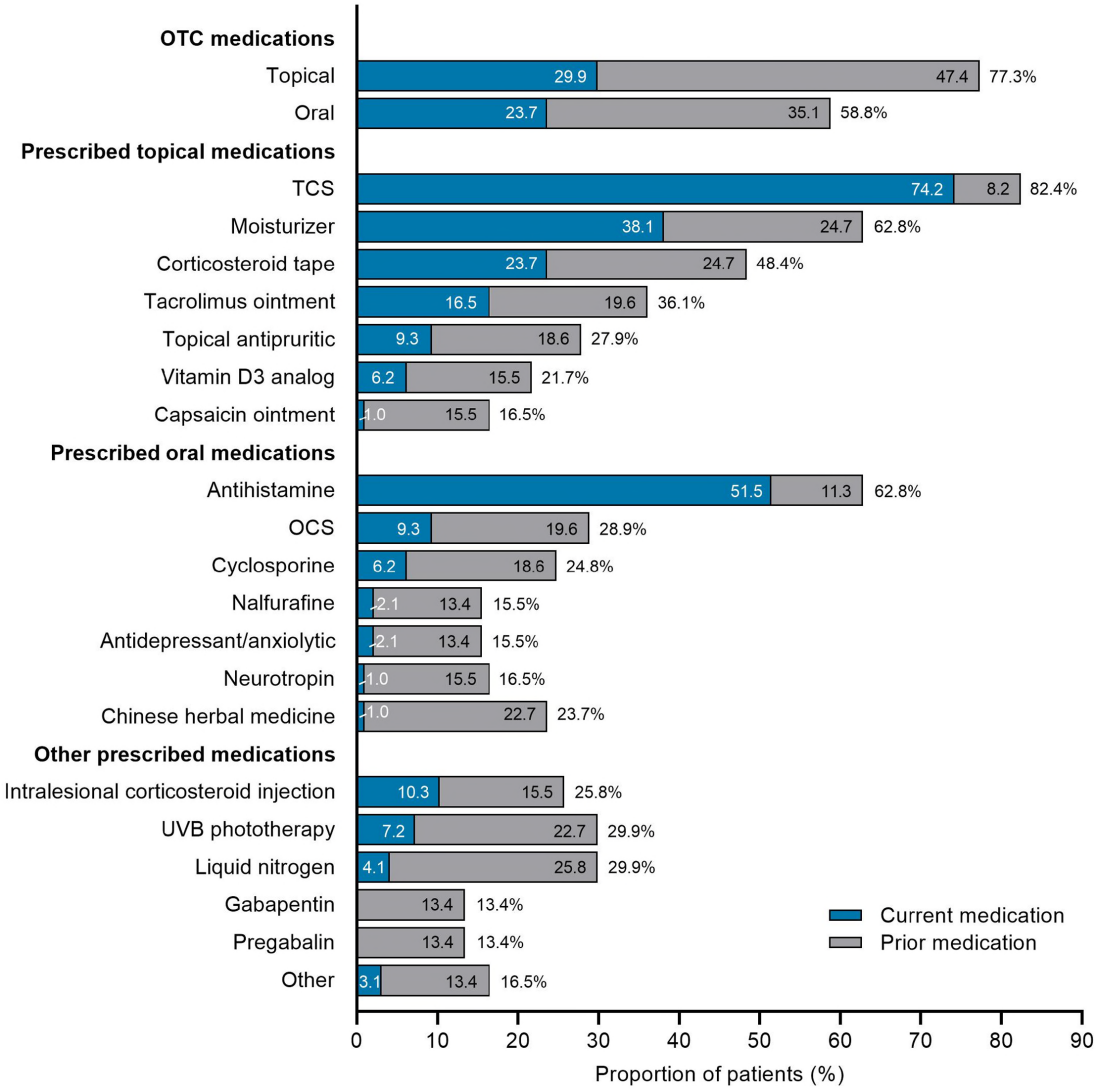
Current and prior medications in patients with prurigo nodularis (*N* = 97). OCS, oral corticosteroid; OTC, over the counter; TCS, topical corticosteroid; UVB, ultraviolet B.

With regard to current routine treatment, the most commonly prescribed medications were TCS (*n* = 72; 74.2%) and oral antihistamines (*n* = 50; 51.5%; Figure 1). OTC topical medications were used by 29.9% of patients (*n* = 29) and OTC oral medications by 23.7% (*n* = 23). When prior and current medication usage were considered, OTC topical medication use was reported by 77.3% of patients (*n* = 75), and OTC oral medication was reported by 58.8% (*n* = 57). By treatment category, 13 patients (13.4%) were receiving OTC medications only, 21 (21.6%) were prescribed TCS therapy (± antihistamines ± UVB phototherapy), 27 (27.8%) were prescribed TCS therapy + adjunctive therapy (as recommended by the 2020 Japanese guidelines),^2^ and 11 (11.3%) were prescribed TCS therapy + systemic OCS therapy or cyclosporine (Table 2). Patient demographics by treatment category are summarized in Table S2. Patients receiving more intensive treatment (i.e., L3 or L4) tended to show a higher prevalence of comorbid AD.

**TABLE 2:** Current treatment category in patients with prurigo nodularis (*N*=97).

Current treatment category, *n* (%)	
L1 OTC skin care only	13 (13.4)
L2 TCS therapy (± antihistamines ± UVB phototherapy)	21 (21.6)
L3 TCS therapy + adjunctive therapy^a^	27 (27.8)
L4 TCS therapy + systemic OCS therapy or cyclosporine	11 (11.3)
Other	25 (25.8)

Abbreviations: L, line; OCS, oral corticosteroid; OTC, over the counter; TCS, topical corticosteroid; UVB, ultraviolet B.

^a^ Includes combined use of local corticosteroid injection, topical heparinoid ointment (occlusive application), vitamin D3 analogs, antipruritic ointment, capsaicin ointment, liquid nitrogen, neurotropin (extracted fluid from the inflamed skin of rabbits inoculated with vaccinia virus), reserpine, gabapentin, pregabalin, or Chinese herbal medicine.^2^

### 3.2 Disease burden

**FIGURE 2:**
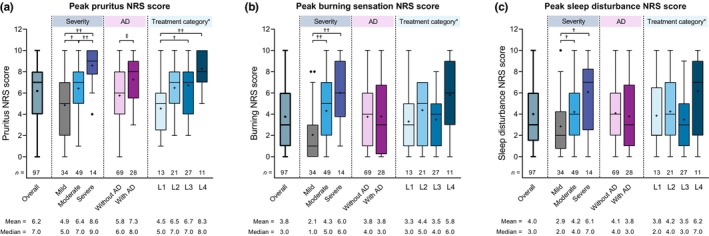
Summary of peak numerical rating scores for (a) pruritus, (b) burning sensation, and (c) sleep disturbance in the overall study population and by disease severity, the presence or absence of comorbid atopic dermatitis, and treatment category. The box depicts first and third quartiles, the horizontal line depicts the median, the whiskers were drawn using Tukey's method; ‘+’ indicates the mean, and black dots indicate outlier values. *Treatment categories were defined as line 1 (L1),  over‐the‐counter (OTC) skin care only; L2, topical corticosteroid (TCS) therapy (± antihistamines ± ultraviolet B [UVB] phototherapy); L3, TCS therapy + adjunctive therapy (as defined by Japanese 2020 treatment guidelines^2^); and L4, TCS therapy + systemic oral corticosteroid (OCS) therapy or cyclosporine. ^†^ *p* < 0.05, ^††^ *p* < 0.01 (Steel–Dwass test); ^‡^ *p* < 0.01 (Mann–Whitney *U* test). SD, standard deviation.

When PN symptoms were evaluated using the NRS, patients had mean±SD peak NRS scores of 6.2 ± 2.7 for pruritus, 3.8 ± 2.9 for burning sensation, and 4.0 ± 2.9 for sleep disturbance (Figure 2). Peak NRS scores increased with increasing disease severity; patients with severe disease had significantly higher peak NRS scores than those with mild disease for pruritus, burning sensation, and sleep disturbance. Patients with comorbid AD had significantly higher peak pruritus NRS scores than those without AD (median 8.0 vs. 6.0), whereas peak burning sensation and sleep disturbance NRS scores were similar between patients with and without AD. Peak pruritus NRS scores were also significantly higher among patients in the most intensive L4 treatment category (i.e., TCS therapy + systemic OCS therapy or cyclosporine) than in those requiring less intensive therapy (median 8.0 vs. 5.0–7.0), whereas peak burning sensation and sleep disturbance NRS scores showed no significant difference across subgroups defined by treatment category.

**FIGURE 3:**
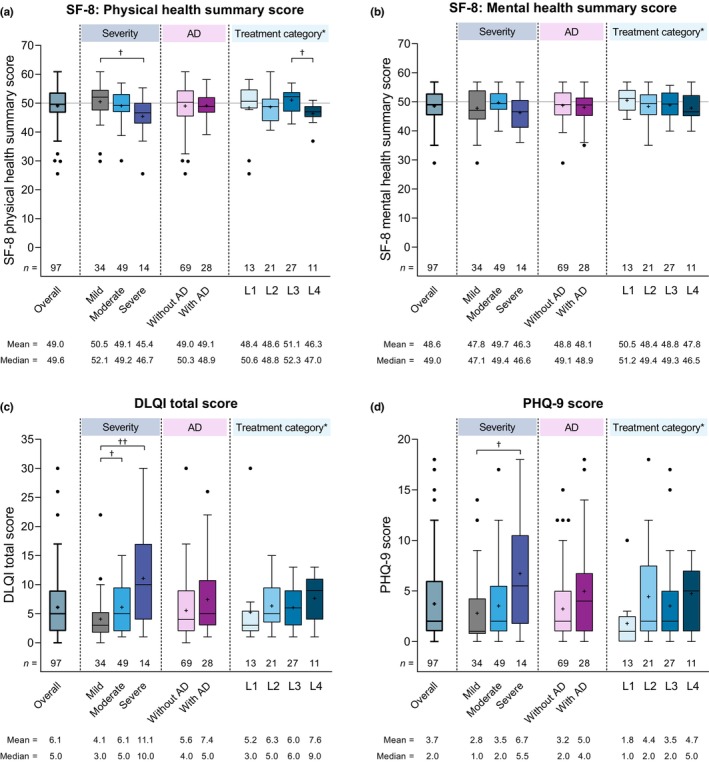
Summary of the (a) Short Form‐8 (SF‐8) physical health summary, (b) SF‐8 mental health summary, (c) total Dermatitis Life Quality Index (DLQI), and (d) Patient Health Questionnaire‐9 (PHQ‐9) scores in the overall study population and by disease severity, the presence or absence of comorbid atopic dermatitis (AD), and treatment category. The box depicts first and third quartiles, the horizontal line depicts the median, the whiskers were drawn using Tukey's method; ‘+’ indicates the mean, and black dots indicates outlier values. The gray horizontal line in (a, b) depicts the Japanese national standard mean SF‐8 score (i.e., 50). *Treatment categories were defined as line 1 (L1), over‐the‐counter (OTC) skin care only; L2, topical corticosteroid (TCS) therapy (±antihistamines ± ultraviolet B [UVB] phototherapy); L3, TCS therapy + adjunctive therapy (as defined by Japanese 2020 treatment guidelines^2^); and L4, TCS therapy + systemic oral corticosteroid (OCS) therapy or cyclosporine. ^†^ *p* < 0.05, ^††^ *p* < 0.01 (Steel–Dwass test). SD, standard deviation.

Regarding HRQoL, patients had median SF‐8 physical and mental health summary scores of 49.6 and 49.0, respectively (Figure 3a,b). The median mental health summary score remained below 50 points (the national standard value in the general Japanese population), regardless of disease severity, while the median physical health summary score for patients with mild or moderate disease was close to or above 50 points and significantly lower in patients with severe versus mild disease. SF‐8 physical and mental health summary scores were generally similar across subgroups defined by the presence or absence of AD or by treatment category except for a significant difference in physical health summary scores between L3 and L4 treatment categories (median 52.3 vs. 47.0).

In addition, DLQI (Figure 3c) and PHQ‐9 (Figure 3d) scores showed gradual worsening with increasing disease severity, with significantly higher DLQI (i.e., worse HRQoL) and PHQ‐9 (i.e., more severe depression) scores in patients with severe versus mild disease, and significantly higher DLQI scores in patients with moderate versus mild disease. However, DLQI and PHQ‐9 scores were not significantly different between patients with and without AD or among the treatment category subgroups.

**FIGURE 4:**
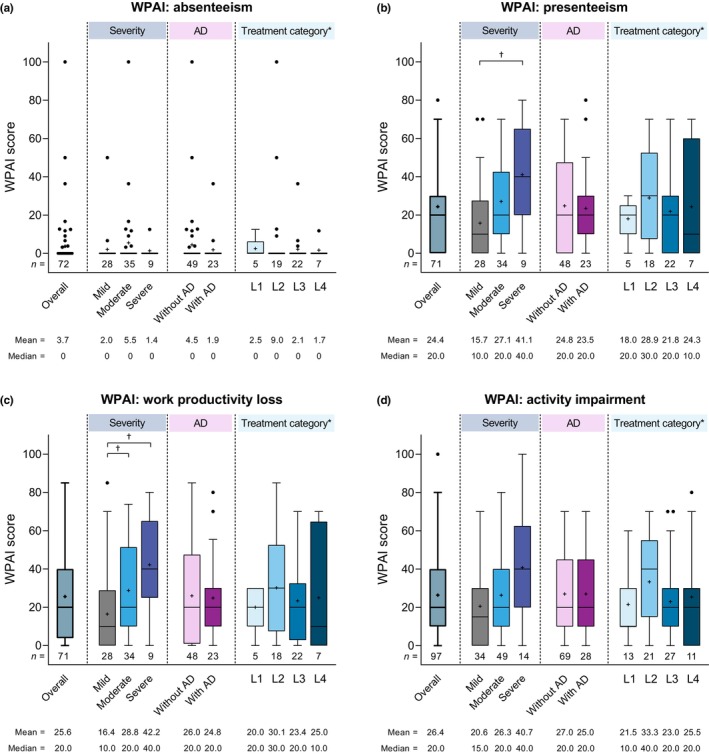
Summary of Work Productivity and Activity Impairment (WPAI) scores for (a) absenteeism, (b) presenteeism, (c) work productivity loss, and (d) activity impairment in the overall study population and by disease severity, the presence or absence of comorbid atopic dermatitis (AD), and treatment category. The box depicts first and third quartiles, the horizontal line depicts the median, the whiskers were drawn using Tukey's method; ‘+’ indicates the mean, and black dots indicate outlier values. *Treatment categories were defined as line 1 (L1), over‐the‐counter (OTC) skin care only; L2, topical corticosteroid (TCS) therapy (±antihistamines ± ultraviolet B [UVB] phototherapy); L3, TCS therapy + adjunctive therapy (as defined by Japanese 2020 treatment guidelines^2^); and L, TCS therapy + systemic oral corticosteroid (OCS) therapy or cyclosporine. ^†^ *p* < 0.05 (Steel–Dwass test). SD, standard deviation.

Significantly increased work‐related impaired performance was observed with increased disease severity with regard to WPAI presenteeism (i.e., work impaired due to PN) and work productivity loss (Figure 4). For example, WPAI presenteeism scores were significantly higher in patients with severe (median 40.0%) versus mild (median 10.0%) disease (*p* = 0.0205), and WPAI work productivity loss scores were also significantly higher in those with severe (median 40.0%) versus mild (median 10.0%) disease (*p* = 0.0176). Although WPAI activity impairment scores showed an increasing trend with increased disease severity, the differences between subgroups were not statistically significant. In contrast, WPAI absenteeism scores remained low across all disease severity subgroups, with no significant difference among groups. WPAI scores across all four domains remained similar in subgroups defined by comorbid AD or treatment category.

### 3.3 Treatment satisfaction

**FIGURE 5:**
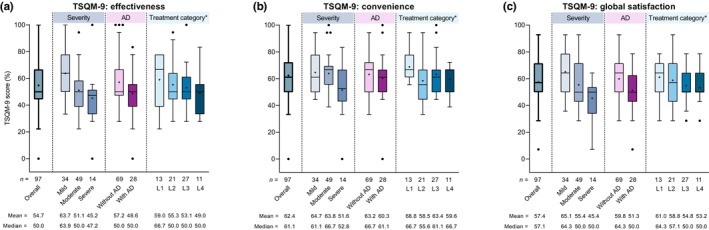
Summary of Treatment Satisfaction Questionnaire for Medication‐9 item (TSQM‐9) scores for (a) effectiveness, (b) convenience, and (c) global satisfaction in the overall study population and by disease severity, the presence or absence of comorbid atopic dermatitis (AD), and treatment category. The box depicts first and third quartiles, the horizontal line depicts the median, the whiskers were drawn using Tukey's method; ‘+’ indicates the mean, and black dots indicate outlier values. *Treatment categories were defined as line 1 (L1), over‐the‐counter (OTC) skin care only; L2, topical corticosteroid (TCS) therapy (± antihistamines ± ultraviolet B [UVB] phototherapy); L3, TCS therapy + adjunctive therapy (as defined by Japanese 2020 treatment guidelines^2^); and L4, TCS therapy + systemic oral corticosteroid (OCS) therapy or cyclosporine. SD, standard deviation.

Regarding current treatment satisfaction, patients had mean±SD TSQM‐9 scores of 54.7 ± 18.1% for effectiveness, 62.4 ± 15.2% for convenience, and 57.4 ± 15.9% for global satisfaction (Figure 5). TSQM‐9 scores for all three domains showed a gradual decrease in treatment satisfaction as disease severity increased, and were lower among patients with comorbid AD than in those without AD. When evaluated by treatment category, mean±SD treatment satisfaction scores for effectiveness, convenience, and global satisfaction were lowest in patients receiving L4 treatment. Convenience scores were higher in patients receiving L1 treatment (i.e., OTC medication only) than in those receiving hospital‐prescribed medications (L2, L3, and L4).

Based on Spearman's rank correlation coefficients, the TSQM‐9 score for global satisfaction showed meaningful negative correlation with GQ score (*r*_s_ = −0.41), peak NRS score for pruritus (*r*_s_ = −0.46), and DLQI total score (*r*_s_ = −0.40); the TSQM‐9 score for effectiveness showed meaningful negative correlation with the peak NRS pruritus score (*r*_s_ = −0.44; Table S3).

**FIGURE 6:**
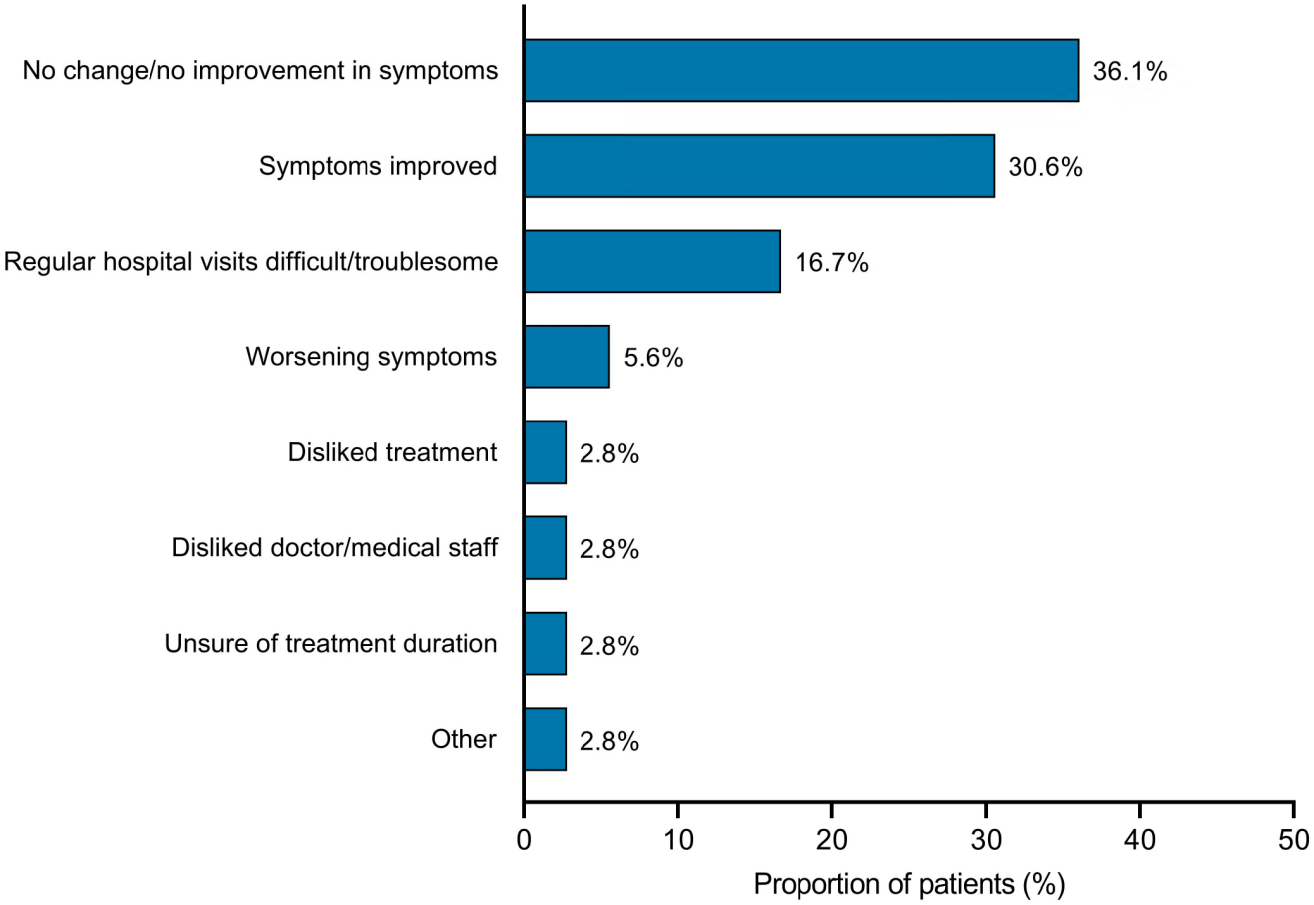
The main reasons for discontinuation of hospital visits in patients with prurigo nodularis (*n* = 36).

In total, 36 patients with PN reported discontinuation of hospital visits, with the main reasons for stopping being no change or no improvement in symptoms (36.1%), or symptom improvement (30.6%; Figure 6).

## 4 DISCUSSION

This cross‐sectional, web‐based survey is the first study to evaluate disease burden and treatment satisfaction among patients with PN in Japan. In this study, increased disease severity based on patient reporting was associated with increased disease burden such as increased pruritus intensity and reduced HRQoL. Current treatment satisfaction also tended to decrease with increased disease severity and did not seem to improve with increased treatment intensity, suggesting an unmet need for alternative therapeutic options for patients with PN.

PN is one of the most pruritic dermatoses. In the current study, weekly peak NRS scores were assessed for pruritus, burning sensation, and sleep disturbance, which are the major symptoms often associated with chronic pruritic skin diseases.^20^, ^21^ All of these symptoms were worsened with increasing disease severity, with pruritus NRS scores being the highest, and was the only symptom to show meaningful negative correlation with TSQM‐9 global satisfaction. This suggests that controlling pruritus might be the key to improving treatment satisfaction in patients with PN.

We used two instruments to describe the potential impact of PN on the patients' HRQoL: the SF‐8 and DLQI. SF‐8 scores can be compared with those of healthy subjects as well as with other diseases,^13^ while the DLQI is specifically designed to assess skin‐disease‐related HRQoL.^14^ While SF‐8 scores showed marginal differences across the severity subgroups, with only the physical health summary score being significantly decreased in patients with severe versus mild disease, DLQI scores showed a clear stepwise impairment of HRQoL (i.e., increase in total scores) with increased disease severity, with significantly higher total scores in patients with moderate versus mild disease and in those with severe versus mild disease. However, the clinical significance of a difference in DLQI scores between those with mild and moderate disease was not clear, given that a DLQI score of 4 indicates a minimal clinically important difference for inflammatory skin diseases.^22^ Similar to the DLQI score, PHQ‐9 scores increased (i.e., depression severity increased) as PN disease severity increased. Patients with severe disease had a significantly higher PHQ‐9 score than those with mild disease (median 5.5 vs. 1.0), which is consistent with these patients having minor to mild depressive symptoms according to the validation studies of PHQ‐9.^15^, ^16^ Previous studies suggested an association between PN, depression, and anxiety.^23^, ^24^ However, as PN severity was not documented in those studies, our current finding suggests for the first time that increased PN disease severity may cause an increased incidence of depressive conditions in these patients. Although 15.5% of the present cohort had previously or were currently receiving antidepressant/anxiolytic drugs, these drugs may be prescribed because of their potential antipruritic effects, as described in the treatment guidelines.^2^ Nevertheless, the potential psychopathological susceptibility of patients with PN must be taken into account on a daily basis of their daily treatment.

Increased PN disease severity was also associated with a significant increase in work‐related impairments with regard to presenteeism (impaired performance) and work productivity based on the WPAI. Of note, the impact on these two domains in patients with severe PN was more than double that observed in those with mild PN. There was also a non‐significant trend towards increased activity impairment with increased disease severity. On the other hand, absenteeism scores remained low or with no missing workdays. These findings suggest that Japanese patients with severe PN are unlikely to miss work, but often have impaired performance and productivity due to their disease burden, confirming the results of a previous report.^25^

In this study, the most prescribed medications (TCS, antihistamines, moisturizers, and corticosteroid tape) were mainly used ‘currently’, while most other guideline‐recommended therapies were reported to have been used ‘previously’ (Figure 1). Of note, OCS and cyclosporine were used by 28.9% and 24.8% of patients, respectively, indicating the refractory nature of PN and a high treatment burden in patients with PN. The long duration of the disease in these patients (median 36 months) may also contribute to accumulated prevalence of OCS and cyclosporine use. Medication use in the current study was generally similar to that reported in Korean and European studies, where TCS and antihistamines were the major medications, and where there was OCS use of >20%.^26^, ^27^ Taken together with our present study, there is a worldwide, unmet medical need for novel effective therapies in patients with severe PN.

It is generally conceivable that the burden of disease is proportionally increased with the number of comorbid diseases. Although AD often coexists with PN,^28^, ^29^ in the current study, comorbid AD did not appear to augment the extent of disease burden, as indicated by the SF‐8, DLQI, PHQ‐9, and TSQM‐9 scores. This suggests that the burden from PN may dominate that from AD; thus, treatment of PN must be prioritized over treatment of AD to alleviate the overall burden of disease in these patients. Future studies are warranted to confirm this hypothesis as the number of patients in this study was limited.

Treatment satisfaction is impacted by multiple factors, such as disease severity, treatment course, and treatment options.^30^ When assessed by treatment category, TSQM‐9 global satisfaction and effectiveness scores showed similar trends, with the lowest scores observed in patients who were receiving the most intensive treatment (i.e., TCS therapy + systemic OCS therapy or cyclosporine). This indicates that the most intensive treatments do not result in high patient‐reported treatment effectiveness, suggesting an unmet need in the management of PN, particularly among patients with severe disease who require L4 treatment with OCS or cyclosporine. Our study findings are in line with those of the European survey of patients with PN, in which treatment satisfaction was reported in only 33.3%, while 28.7% considered that none of the treatment options were effective.^10^ Therapeutic options that may improve treatment satisfaction are especially important for patients with severe PN, who may also experience anxiety and depressive symptoms.^8^ In the present study, GQ, peak pruritus NRS, and DLQI showed meaningful correlation with treatment satisfaction, suggesting the need for effective measures for pruritus and related HRQoL to improve treatment satisfaction, as previously reported for AD.^25^, ^31^ Indeed, several novel therapeutics are now under development, and future studies of the real‐world effectiveness and safety of these agents are anticipated.^32^, ^33^

Limitations of this study include the possible influence of selection bias on the results, as dermatologists could choose whether they invited their patients, and patients could choose whether they responded to the questionnaire. In addition, as all study outcomes were based on PROs, the data may be affected by recall bias. Lastly, due to the cross‐sectional nature of this study, no causal inferences can be made regarding the correlations between treatment satisfaction and PROs.

In summary, this web‐based questionnaire of patients with PN in Japan found that the disease burden increased with increasing PN disease severity. Increased disease severity and increased treatment intensity appeared to be associated with reduced treatment satisfaction. More effective treatment options are needed to reduce disease burden and improve treatment satisfaction among patients with currently intractable severe PN.

## FUNDING INFORMATION

This study and editorial assistance for the preparation of this article was supported by Sanofi K.K. The funding source was involved in the study design, data interpretation, and manuscript preparation.

## CONFLICT OF INTEREST STATEMENT

HM has received lecture fees from Sanofi K.K., AbbVie GK, Eli Lilly Japan K.K., and Maruho Co., Ltd. KA, TY, and HF are Sanofi K.K. employees and may hold stock and/or stock options in the company.

## Supporting information


Table S1.

Table S2.

Table S3.

## Data Availability

Qualified researchers may request access to patient‐level data and related documents (including, e.g., the clinical study report, study protocol with any amendments, blank case report form, statistical analysis plan, and dataset specifications). Patient‐level data will be anonymized, and study documents will be redacted to protect the privacy of trial participants. Further details on Sanofi's data sharing criteria, eligible studies, and process for requesting access can be found at https://www.vivli.org/.
