# Supplementary material for: Disease burden and treatment satisfaction in patients with prurigo nodularis in Japan
Source: J Dermatol. 2023 Dec 8;51(2):223–33. doi: 10.1111/1346-8138.17045 (PMC11484124; doi:10.1111/1346-8138.17045)
Supplement: Supplementary file 1 — Table S1. [file JDE-51--s002.docx]

**SUPPORTING TABLE S1** Patient demographics and baseline characteristics by presence of comorbid atopic dermatitis

| **Item** | **Comorbid AD** | |
| --- | --- | --- |
|  | **Without AD (n=69)** | **With AD (n=28)** |
| Sex, n (%) |  |  |
| Male | 37 (53.6) | 17 (60.7) |
| Female | 29 (42.0) | 11 (39.3) |
| Unknown/no answer | 3 (4.3) | 0 |
| Mean ± SD age, years | 53.9 ± 12.4 | 47.8 ± 13.5 |
| Mean ± SD PN duration, months | 50.5 ± 54.5 | 107.6 ± 126.5 |
| Mean ± SD GQ score | 1.8 ± 0.8 | 2.0 ± 0.9 |
| Disease severity by GQ score, n (%) |  |  |
| Mild (GQ score = 0–1) | 26 (37.7) | 8 (28.6) |
| Moderate (GQ score = 2) | 33 (47.8) | 16 (57.1) |
| Severe (GQ score = 3–4) | 10 (14.5) | 4 (14.3) |

Abbreviations: AD, atopic dermatitis; GQ, Global Question; PN, prurigo nodularis; SD, standard deviation
