# Supplementary material for: Disease burden and treatment satisfaction in patients with prurigo nodularis in Japan
Source: J Dermatol. 2023 Dec 8;51(2):223–33. doi: 10.1111/1346-8138.17045 (PMC11484124; doi:10.1111/1346-8138.17045)
Supplement: Supplementary file 2 — Table S2. [file JDE-51--s003.docx]

**SUPPORTING TABLE S2** Patient demographics and baseline characteristics by treatment category

| **Item** | **Treatment category** | | | |
| --- | --- | --- | --- | --- |
|  | **L1 = OTC skin care only (n=13)** | **L2 = TCS therapy^†^ (n=21)** | **L3 = TCS therapy^†^ + adjunctive therapy^‡^** **(n=27)** | **L4 = TCS therapy^†^ + systemic OCS therapy or cyclosporine (n=11)** |
| Sex, n (%) |  |  |  |  |
| Male | 6 (46.2) | 11 (52.4) | 16 (59.3) | 6 (54.5) |
| Female | 4 (30.8) | 10 (47.6) | 11 (40.7) | 5 (45.5) |
| Unknown/no answer | 3 (23.1) | 0 | 0 | 0 |
| Mean ± SD age, years | 51.6 ± 14.9 | 45.7 ± 12.5 | 51.6 ± 12.9 | 53.8 ± 14.1 |
| Mean ± SD PN duration, months | 72.5 ± 109.5 | 32.3 ± 27.5 | 66.1 ± 54.1 | 101.9 ± 123.4 |
| Mean ± SD GQ score | 1.7 ± 0.6 | 1.7 ± 0.6 | 1.9 ± 0.6 | 2.2 ± 1.0 |
| Comorbid AD, n (%) | 3 (23.1) | 4 (19.0) | 11 (40.7) | 5 (45.5) |

Abbreviations: AD, atopic dermatitis; GQ, Global Question; L, line; OCS, oral corticosteroid; OTC, over-the-counter; PN, prurigo nodularis; SD, standard deviation; TCS, topical corticosteroid; UVB, ultraviolet B
^†^With or without antihistamines or UVB phototherapy^2^
^‡^Includes the following options: local corticosteroid injection, topical heparinoid ointment (occlusive application), vitamin D3 analogues, tacrolimus ointment, antipruritic ointment, capsaicin ointment, liquid nitrogen, neurotropin (extracted fluid from the inflamed skin of rabbits inoculated with vaccinia virus), reserpine, gabapentin, pregabalin, or Chinese herbal medicine^2^
