# Supplementary material for: Disease burden and treatment satisfaction in patients with prurigo nodularis in Japan
Source: J Dermatol. 2023 Dec 8;51(2):223–33. doi: 10.1111/1346-8138.17045 (PMC11484124; doi:10.1111/1346-8138.17045)
Supplement: Supplementary file 3 — Table S3. [file JDE-51--s001.docx]

**SUPPORTING TABLE S3** Correlation between TSQM-9 scores and patient-reported outcomes

|  | **TSQM-9** | | | | | |
| --- | --- | --- | --- | --- | --- | --- |
|  | **Effectiveness** | | **Convenience** | | **Global satisfaction** | |
|  | **r_s_** | **p** | **r_s_** | **p** | **r_s_** | **p** |
| GQ (n=97) | –0.38 | <0.001 | –0.18 | 0.0845 | –0.41 | <0.001 |
| Maximum NRS score (n=97) |  |  |  |  |  |  |
| Pruritus | –0.44 | <0.001 | –0.37 | <0.001 | –0.46 | <0.001 |
| Burning sensation | –0.26 | 0.0088 | –0.32 | 0.0012 | –0.33 | <0.001 |
| Sleep disturbance | –0.28 | 0.0058 | –0.23 | 0.0227 | –0.19 | 0.0557 |
| SF-8 (n=97) |  |  |  |  |  |  |
| Physical health summary score | +0.28 | 0.0055 | +0.26 | 0.0095 | +0.37 | <0.001 |
| Mental health summary score | +0.10 | 0.3354 | +0.11 | 0.2920 | +0.08 | 0.4173 |
| DLQI total score (n=97) | –0.36 | <0.001 | –0.33 | <0.001 | –0.40 | <0.001 |
| PHQ-9 (n=97) | –0.07 | 0.4775 | –0.19 | 0.0576 | –0.17 | 0.0911 |
| WPAI |  |  |  |  |  |  |
| Absenteeism (n=72) | 0.03 | 0.8305 | –0.12 | 0.3141 | –0.12 | 0.3208 |
| Presenteeism (n=71) | –0.14 | 0.2537 | –0.23 | 0.0488 | –0.16 | 0.1833 |
| Work productivity loss (n=71) | –0.14 | 0.2549 | –0.25 | 0.0370 | –0.18 | 0.1275 |
| Activity impairment (n=97) | –0.14 | 0.1745 | –0.34 | <0.001 | –0.12 | 0.2277 |

Abbreviations: DLQI, Dermatology Life Quality Index; GQ, Global Question; NRS, Numerical Rating Scale; PHQ-9, Patient Health Questionnaire 9; r_s_, Spearman’s rank correlation coefficient; SF-8, Short Form 8; TSQM-9, Treatment Satisfaction Questionnaire for Medication – 9 items; WPAI, Work Productivity and Activity Impairment.
